# Inconsistent subthalamic local field potential beta activity amid in- and antiphasic neuronal bursts

**DOI:** 10.1038/s41531-026-01531-4

**Published:** 2026-09-16

**Authors:** Maximilian Scherer, Nina Wiedemann, Kai Bötzel, Matthias Löhle, Thomas Kriesen, Daniel Cantré, Jan Hinnerk Mehrkens, René Reese, Thomas Koeglsperger

**Affiliations:** 1 Department of Neurology, LMU University Hospital, LMU Munich, Munich, Germany; 2 Department of Neurology, Rostock University Medical Center, Rostock, Germany; 3 German Center for Neurodegenerative Diseases (DZNE) Rostock/Greifswald, Rostock, Germany; 4 Department of Neurosurgery, Rostock University Medical Center, Rostock, Germany; 5 Institute and Policlinic of Radiology, Pediatric Radiology and Neuroradiology, Rostock University Medical Center, Rostock, Germany; 6 Department of Neurosurgery, LMU University Hospital, LMU Munich, Munich, Germany; 7 Department of Translational Brain Research, German Center for Neurodegenerative Diseases (DZNE), Munich, Germany

**Keywords:** Biomarkers, Neurology, Neuroscience

## Abstract

Elevated subthalamic (STN) beta (12–32 Hz) local field potentials (LFP) are a hallmark of Parkinson’s disease and closely tied to synchronized spiking. The biomarker guides deep brain stimulation (DBS) lead implantation and is under investigation to inform DBS programming and adaptive DBS, yet its prevalence has not been independently assessed. This study assesses the biomarkers prevalence and investigating its relationship to synchronized beta-bursting neurons. STN LFPs and spiking activity of *n* = 156 patients with Parkinson’s disease from seven DBS centers recorded via microelectrodes were examined and spectral LFP peaks classified. The distribution of phase angles between beta LFP and beta-bursting neurons was explored. Beta LFPs were bilaterally expressed in 47.25% of patients (65.59% of hemispheres). LFP-synchronized neuronal spiking clustered in two peaks, 182.84° phase shifted. This applied on the group level and for individual patients. Beta-LFP-informed approaches mandate reliable biomarker expression, a requirement not met by 52.75% of patients. This is particularly troublesome for intraoperative guidance wherein the cause of biomarker absence, including DBS lead misplacement or a balanced ratio of phasic and antiphasic spiking neurons, cannot be determined. While STN-LFP beta remains a valuable biomarker, multiple biomarkers should be evaluated simultaneously to increase robustness.

## Introduction

Increased subthalamic (STN) local field potential (LFP) oscillations in the beta frequency range (12–32 Hz) are considered a hallmark of Parkinson’s disease^1^, closely related to hypokinetic motor impairment. Suppression of STN-LFP beta via deep brain stimulation (DBS) accompanies symptomatic improvement^2^, prompting widespread adoption of beta as a biomarker to guide electrode implantation^3–8^, programming^9–11^, and adaptive DBS (aDBS)^12^. However, clinical performance of beta-informed strategies is inconsistent, and a substantial proportion of patients don’t express beta^3,9^. Reported per-hemisphere beta prevalence ranges from 64 to 69.5%^2,9,13^ in small cohorts to 84.8% (64.2% bilateral; *n* = 51) in the ADAPT-PD trial^14^ and 89.0% (69.4% bilateral; *n* = 113)^15^ in Medtronic’s BrainSense product surveillance registry (BrainSense PSR), a difference of ~20%. However, differences in cohort selection and beta definition across studies complicate direct comparison of these estimates. Consequently, the true prevalence and generalizability of STN-LFP beta activity across unselected Parkinson’s disease populations remain uncertain.

The absence of beta has direct clinical implications. Electrophysiological mapping supplements continuously improving anatomical targeting^16^, which is limited by imaging resolution^17,18^ and brainshift^19,20^. Although beta LFPs offer a simpler alternative^21^ to 1998’s notoriously difficult to interpret^22^ gold-standard of microelectrode based guidance^23^, their utility depends on bilateral STN-LFP beta expression. Consequently, this added dependency may complicate interpretation when STN-LFP beta activity is absent. For example, MER recordings or acute clinical testing may indicate appropriate lead placement, while the concurrent lack of a detectable beta peak may create ambiguity in the interpretation of biomarker signals. This is clinically relevant as improper lead placement and insufficient therapeutic response remain important challenges, with 15.2–34.0% of implantations required revision or removal in North America from 2004 to 2013. 48.5% of these cases were attributed by Rolston et al. to potentially improper lead placement or insufficient therapeutic response^24^. In a separate investigation of suboptimal DBS outcomes, Okun et al. attributed 46% of insufficient therapeutic responses to DBS lead misplacement^25^. Similar limitations affect guided DBS programming and aDBS: Beta informed approaches fail to identify clinically selected contacts in 8–50% of people^3,9,26^, show low co-variability (17.30%) with core motor symptoms, rigidity and bradykinesia^27^, and are not deployable in patients without beta expression^28^. Together with sporadic reports of the biomarker’s absence, i.e. Tinkhauser^9^ et al. reporting 3 out of 8 patients and Binder et al.^29^ reporting 7 out of 19 hemispheres without detectable beta, and a strong publication bias (*p* < 0.006) favoring positive outcomes^30^, these observations indicate beta prevalence may present a practical limitation for beta-informed systems^12,31^.

The absence of STN-LFP beta in some patients remains unresolved. Beta LFPs have been shown to co-occur with beta-bursting neuronal spiking in the STN^1,23^ in humans, with spiking activity of beta-bursting neurons^32^, particularly spiking within-bursts^33^ being synchronized to the LFP. However, pathological spiking and LFPs have been dissociated in animal models^34,35^ with spiking’s contribution outweighing oscillations’ impact on motor control^34^. While it is assumed that spiking may directly or indirectly contribute to LFP activity^36^, a causal explanation permitting beta-bursting neurons in the absence of detectable beta LFPs has not yet been established.

Here, we quantify the prevalence of elevated STN-LFP beta power in 156 patients across seven DBS centers and analyze single-neuron activity (606 units) to determine whether phase relationships among beta-bursting neurons account for the absence of detectable beta LFPs.

## Results

### Bilateral prevalence of STN-LFP beta power is 47.25% per patient

**Fig. 1:**
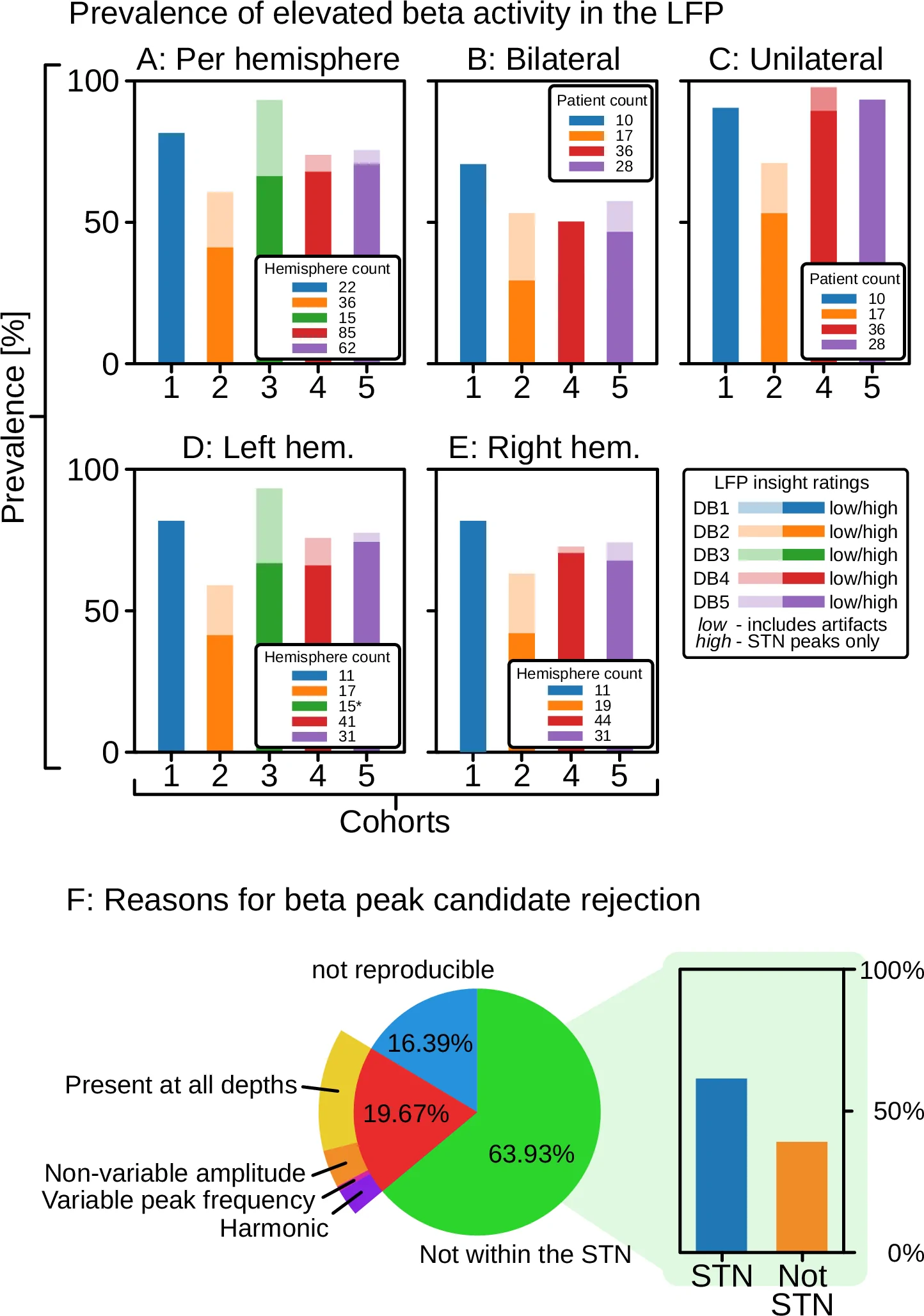
Prevalence of elevated beta band local field potentials (LFPs). Prevalence of elevated beta band local field potentials (LFPs) in the subthalamic nucleus (STN) of people with Parkinson’s disease, per hemisphere (**A**; 65.59%), bilaterally (**B**; 47.25%), unilaterally (**C**; 83.51%), in the left hemisphere (**D**; 66.08%), and the right hemisphere (**E**; 65.71%), separated into counts without (low insight; partially transparent) and with (high insight; opaque) artifact rejection. Beta peak candidates were rejected (**F**) for being outside the STN (63.93%), being not reproducible (16.39%), or another type of artifact (16.67%).

To analyze the prevalence of elevated STN-LFP beta power, data acquired from extra- (cohorts 1 and 2) and intraoperative recordings (cohorts 3–5) were analyzed. Without artifact rejection, per-hemisphere prevalence was 74.54% (average; see Fig. 1A: partially transparent *low insight* bars). Post artifact rejection, per-hemisphere STN-LFP beta prevalence was 65.59% (average; see Fig. 1A: colored *high insight* bars). These results were mostly consistent across all cohorts, with cohort 1 having the highest and cohort 2 the lowest prevalence. Bilateral prevalence (left and right hemisphere) of STN-LFP beta was 47.25% per patient (average; see Fig. 1B) when rejecting artifacts 54.94% without artifact rejection. Unilateral prevalence (left or right hemisphere, or both) of STN-LFP beta without artifact rejection was 90.10% (average; see Fig. 1C), 83.51% after artifact rejection. Comparing STN-LFP beta power prevalence between the left (Fig. 1D) and right hemisphere (Fig. 1E) did not reveal any systematic differences. Of note, as data from cohort 3 could not be attributed toward a specific hemisphere, data is visualized in left-hemisphere plots (see Fig. 1A, D). Any beta peak candidates identified as artifacts were classified as such for the following reasons: Beta peaks were post-hoc localized as not within the STN (63.93%; cohorts 4 & 5 only; see Fig. 1F). They were not reproducible (16.39%; cohorts 3–5 only). They were another type of artifact, including persistent effects and harmonics (19.67%).

Finally, alpha activity was less prevalent (as expected), presenting a far more sporadic expression (see Supplementary Fig. 5).

### Beta bursting neurons are phase locked to STN-LFP activity at two opposing angles

**Fig. 2:**
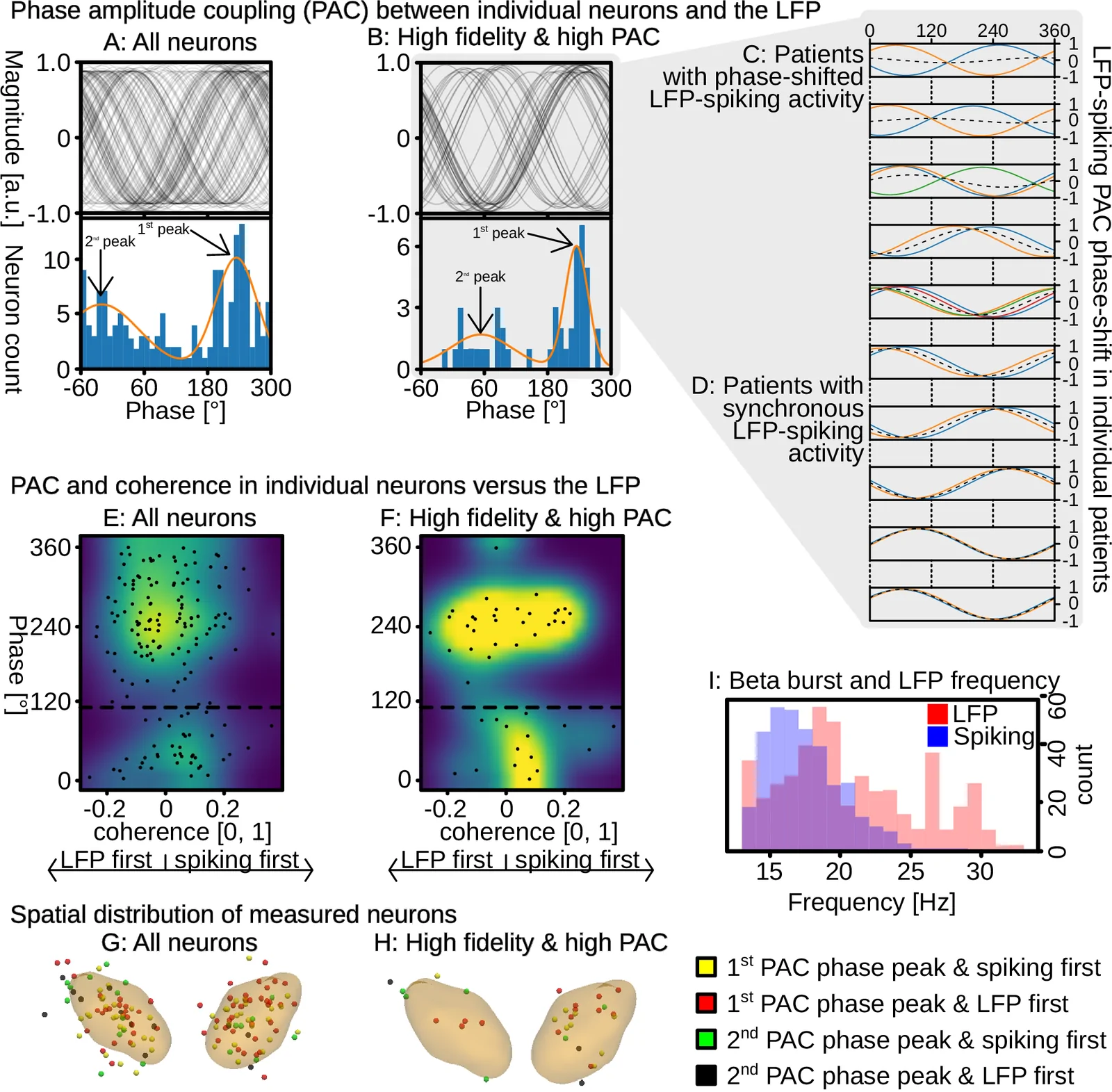
Analysis of single-neuron spiking to local field potential (LFP) coupling. Herein, we employed the direct modulation index^57^ to measure coupling between neuronal spiking and the LFP as this metric projects spiking onto a limited scale with ‘*0*’ indicating no coupling and ‘*1*’ indicating full coupling. First, we computed the sinusoidal fit between the spiking activity and the LFP across all neurons (**A**; upper panel) and for high fidelity neurons with a high phase-amplitude coupling (PAC) score (**B**; upper panel). Next, the phase difference between the spiking and LFP activity was aggregated, revealing two clusters at 234.50° and −20.18° across all neurons (**A**; lower panel) and 235.19° and 52.35° for high fidelity, high PAC neurons (**B**; lower panel). Subsequently, we verified the present of non-phase-aligned (**C**) and phase-align neurons (**D**) across individual patients. An estimate of the additively combined activity profile across all neurons is given as the black dashed line. Further analyses revealed a difference in coherence between these two populations of neurons. Namely, neurons clustering at the 1st peak did not exhibit a significant preference whether the spiking or LFP occurred first (**E** & **F** – upper), yet spiking of the 2nd peak population mostly preceded the LFP (**E** & **F** – lower). A spatial difference between these two populations of neurons was not observed (**G** & **H**). Beta peaks for enveloped spiking activity and LFP were similar (**I**).

Having previously observed a strong coupling between within-burst spiking activity of single neurons and LFPs^33^, this relationship was further explored by quantifying the phase angle homogeneity between the two electrophysiological phenomena. Including all neurons, activity synchronized to the LFP at 234.50° (Fig. 2A) with a secondary population at −20.18°, yielding a phase difference of 254.68°. When focusing on high fidelity, high PAC neurons, peaks shift to 235.19° and 52.35°, with a phase difference of 182.84°. This observation was replicated across individual patients across all applicable cohorts (3, 4, and 5; Fig. 2C, D). Calculating an artificial additive composite signal (black striped line) of each patient’s neurons demonstrated that phase shifted neurons result in low amplitude signal (Fig. 2C) whereas phase synchronized ones result in high amplitude signal (Fig. 2D).

Further analysis revealed that these populations do not only differ in their phase angle to the LFP, but also their directionality to the LFP. Namely, while there is no distinct directional sequence (*p* < 0.06; 56 vs. 64 neurons) between the LFP and the enveloped bursting signal of 1st peak neurons (234.68°; Fig. 2E, upper point cloud), neuronal spiking did precede (*p* < 0.02; 15 vs. 29 neurons) the LFP for the 2nd peak (−20.18°; Fig. 2E, lower point cloud). Similarly, there was no distinct directional sequence (*p* < 0.12; 13 vs. 17 neurons) when focusing only on the enveloped bursting signal of high fidelity, high PAC 1st peak neurons (235.19°; Fig. 2F, upper point cloud). Yet, neuronal spiking again preceded (*p* < 0.0418; 4 vs. 11 neurons) the LFP for the 2nd peak (52.35°; 2 F, lower point cloud). Analyzing the spatial distribution of these neurons (Fig. 2G, H) did not reveal any succinct patterns.

Finally, comparing frequency distributions of the LFP and spiking activity revealed similar peaks (Fig. 2I).

## Discussion

Electrophysiological analyses are increasingly employed in daily medical routine, however, the prevalence of underlying biomarkers often remains insufficiently investigated. Herein, we explored the prevalence of subthalamic (STN) local field potentials (LFPs) in the beta frequency band (13–32 Hz), considered a hallmark in people with Parkinson’s disease^1^. This biomarker may be used to guide various steps in DBS treatment, hence requiring reliable bilateral biomarker expression to be applicable or perform optimally. However, herein we found that this biomarker is only expressed in slightly less than every other patient bilaterally (47.25%). Exploring the relationship between neuronal spiking activity and LFPs provided a potential explanation. Namely, we observed two distinct populations of neurons, being coupled to the LFP at 52.35° and 235.19°. While the exact relationship between spiking and LFP remains elusive, the herein observed partially phasic-antiphasic relationship of neurons to the LFP may be disadvantageous for LFP production. Furthermore, these two populations also expressed varying directional relationships to the LFP. Namely, the secondary population was significantly tilted toward acting prior to the LFP, whereas the main population did not express any observable preference, supporting the identification as two separate populations.

The herein observed ratio of STN-LFP beta expression, 47.25%, is lower than those reported in both the ADAPT-PD trial (64.0%)^14^ and the BrainSense PSR (69.4%)^15^, yet had similar per-hemisphere results as earlier small scale reports^2,9,13^ (64–69.5% vs. 65.26%). These differences may reflect a number of potential reasons.

First, varying definitions of beta activity may have contributed to this difference with the ADAPT-PD and PSR trials including alpha (8–12 Hz) in the beta range. However, given the largely inconsistent pattern of alpha activity observed herein, this aspect is unlikely to be the sole cause. An alternative explanation may lie in trial-specific differences. Namely, ADAPT-PD prescreened an undisclosed number of patients for beta and PSR potentially treated the contact with the highest average power as a beta peak. Beyond these systematic differences, extra-subthalamic beta peaks may have also been inadvertently misclassified as STN-LFP beta.

Second, the observation of such beta peaks in the substantia nigra pars reticula (SNr) was common herein and in previous reports^37,38^. Hence, beta peaks, particularly at the end of DBS electrodes or in deeper implanted DBS leads may have erroneously been misclassified in cases where not all electrode positions had been post-hoc reconstructed to verify proper positioning. Likewise, approximately one in six beta peaks observed herein were not reproducible as these appeared in a single measurement and never thereafter. Hence, it is conceivable that noise-like artifacts may have inflated beta prevalence further.

Third, as biomarkers are not reliably expressed across all patients, sampling bias may also partially explain differences in biomarker expression rates. For example, the smaller cohorts investigated herein (#1 and #2) expressed much more variability in terms of STN-LFP beta expression than the two to four times larger cohorts from Munich and Rostock (#4 and #5).

The herein observed limited prevalence of bilateral beta STN-LFP expression limits the biomarkers applicability to guide DBS lead implantation. Previously, the region of strongest beta elevation has repeatedly been observed to co-localize with the optimal DBS lead implantation site within the STN^3,39^ and correlate significantly with symptoms^22^. As post-hoc analyses have demonstrated, the optimal implantation trajectory is often marked by strong beta expression^3^, in up to 80% of cases^4^, the biomarker has been suggested as a reliable indicator of proper lead placement^40^. Conversely, beta activity was only observed in 65.59% (vs. 64–69.5% small scale reports^9,13,41^) of hemispheres with bilateral prevalence at only 47.25%, slightly less than every other patient. While the above discussed causes may technically provide an answer to this discrepancy (i.e. SNr beta and/or increased broadband activity within nuclei), intraoperative information is usually too limited to determine the cause of biomarker (non-)expression. Namely, an increase of beta LFPs may also be observed when entering any nucleus (increase in broadband power), entering the STN or SNr specifically, or due to noise forming a non-reproducible beta peak. Likewise, absence of beta LFPs may be observed when misplacing DBS leads or in patients without biomarker non-expression (34.41% of hemispheres). Hence, it may be difficult to select the optimal trajectory from beta LFPs alone. While beta LFPs may provide important additional insights, it is advisable to corroborate these with microelectrode recordings and/or imaging information.

Beyond guiding DBS lead implantation, STN-LFP beta power is currently under investigation to guide DBS programming and adaptive DBS, as STN-LFP beta is considered a reliable biomarker^40^ to assess DBS-related treatment benefits^2^. As such, it has been selected to guide several manual and (half-)automated DBS programming (i.e. Medtronic BrainSense^15^), and adaptive DBS (aDBS) approaches. Yet, while significantly correlating with symptom expression^22^, LFP variability only poorly captures symptom severity, indicated by qualitatively weak correlation (r² ≈ 17%)^27^. Correspondingly, while initial assessments of beta informed aDBS had been very positive, albeit with a significant publication bias toward positive outcomes^30^, recent trials’ outcomes are more modest^42^, with improved ON-time over continuous DBS in 36 cases (52.94%) and no impact or worsened in 32 cases (47.06%)^43^. The often exclusive use of beta^12^ limits applicability to patients with at least unilateral biomarker expression, as demonstrated by Oehrn et al.^28^. Yet, it is assumed that bilateral sensing is required for optimal aDBS performance^44^. However, this condition is not met by 52.75% of patients. A potential way forward may lie in biomarker diversification, whereby sets of features may increase prediction performance and provide redundancy, thereby strengthening support for biomarker-based methods. To this end, several additional biomarkers may be explored, including beta-gamma coupling^28,45^, finely tuned gamma oscillations^46^, and cortico-cortical beta-band coupling^47^. While some of these biomarkers, such as finely tuned gamma oscillations, are independent of STN-LFP beta activity, their prevalence and informational overlap with STN-LFP beta has not yet been established.

While the exact relationship between single neuron and conglomerate multi-unit activity, such as LFPs, has not yet been determined, it is assumed that LFPs may constitute at least in part from synaptic currents and synchronized spiking activity^36^. Spiking activity has been shown to synchronize at a specific phase of the LFP^32^, especially when occurring within bursts^33^. Herein, this concept was expanded by characterizing the phase-angle between spiking and LFP activity. While most neurons’ spiking synchronized at 235.19°, with one-third of neurons’ spiking synchronized at 52.35°, a 182.84° phase shift. This result was repeated across individual patients from all three applicable cohorts investigated herein. Conceptually, activity of this antiphasic minority may dampen overall LFP activity levels, which are measured by a macroelectrode or sensed from a DBS lead. Namely, as contributions from individual neurons likely average in multi-unit LFP activity^36^, individual neuron’s activity must be aligned to meaningfully contribute to the LFP. However, if phasic and antiphasic, the sum of individual neuron’s activity would negate on a macroscopic scale, with a continuous effect on the LFP magnitude between aligned and phasic/antiphasic scenarios. While the ratio of in-phase and antiphasic neurons within an individual human subject cannot be determined at this point, the herein established concurrent presence of neurons from both populations within the same patients provide a potential causal explanation for reduced or absent beta activity in the STN-LFP – given the assumption that spiking significantly contributes to the LFP^36^.

If confirmed in future studies, this observation may provide a mechanistic explanation for the absence or attenuation of beta-band STN-LFP activity in a subset of patients, partially explaining the electrophysiological heterogeneity of the indication. Specifically, varying proportions of phasic and antiphasic neuronal populations could reduce the net macroscopic beta signal despite the presence of beta bursting activity. Such a mechanism would have important implications for biomarker-guided DBS approaches, as absence of a detectable beta peak may not necessarily indicate suboptimal lead placement or absence of pathophysiological beta synchronization at the neuronal level.

While having assessed STN-LFP beta prevalence without immediate subsequent use, several limitations apply to this work. Namely, the low-frequency attenuation introduced by low-order hardware filters in cohorts 4 and 5 was reversed through inverse filtering to recover the raw signals. Albeit the application of a filter is a simple multiplication in the frequency domain, an easily reservable step, this may have introduced additional noise, as does most data processing. However, the original signal was numerically stable, and correct filter reversal was manually confirmed (see Supplementary Fig. 1) supported by the close similarity of results across cohorts. For cohorts 4 and 5, only the final implantation trajectory was analyzed in patients with multiple trajectories. While DBS lead positioning and microelectrode trajectory projections were verified via anatomical reconstruction and visual confirmation, anatomical inaccuracies may have impacted beta estimates. To compensate, beta peaks within 1 mm of the STN were still considered inside the STN. While anesthesia can influence beta activity^48^, no recordings were obtained during active anesthesia. Cohorts 3 and 5 were acquired under local anesthesia, whereas cohorts 4 was obtained predominantly under general anesthesia that was discontinued at least 10 min before recording; nevertheless, bilateral and unilateral beta prevalence was comparable across centers. Postoperative microlesion effects^49^ may have increased noise^50^ in cohorts 1 and 2, but their combined results remained consistent with intraoperative cohorts (3–5).

Finally, albeit bilateral STN-LFP beta expression was observed in only 47.25% of patients, the biomarker offers valuable information that can be leveraged for patient benefit. However, given its limited prevalence, additional biomarkers such as beta-gamma coupling, finely tuned gamma, and/or cortico-cortical beta-band coupling may be assessed in parallel to increase robustness. This approach allows for reliable estimates – such as confirming proper DBS lead placement when a single biomarker is not expressed.

## Methods

### Patients

Data were pooled from publicly available sources^33,46,51^ and collaboration partners in Munich and Rostock, Germany, in total data from seven DBS centers grouped into five cohorts were combined. Table 1 provides an overview of the employed data. Dataset 1 comprises multi-center (*n*_center_ = 3) recordings of 16 patients from London, UK and Mainz, DE^46^. Dataset 2 comprises recordings of 19 patients from Düsseldorf, DE^51^. Dataset 3 comprises recordings of 15 patients from Toronto, CA^33^. Dataset 4 comprises recordings of 67 patients from Munich, DE. Dataset 5 comprises recordings of 39 patients from Rostock, DE. Data distribution/local collection and evaluation have been approved by the ethics committee’s of the LMU and the Rostock University Medical Center, and written informed consent has been obtained from all study participants in accordance with the declaration of Helsinki.

**Table 1:** Overview on data acquired from various sources. Only data OFF medication and OFF DBS was assessed

ID	Public data	Patients	LFP traject-ories	Micro-electrode recordings	Intra- operative	Post- operative	DBS center	Data acquisition
1	Yes	16	32	–	–	3–6 days	St. George’s University (London, UK), King’s College Hospital (London, UK) University Medical Center Mainz (Mainz, DE)	n/a
2	Yes	19	38	–	–	1 day	Heinrich Heine University (Düsseldorf, DE)	n/a
3	Yes	15	15	97	Yes	–	Toronto Western (Toronto, CA)	n/a
4	No	67	134	3995	Yes	–	LMU Clinic (Munich, DE)	2017–2026
5	No	39	78	5072	Yes	–	Rostock Medical Center (Rostock, DE)	2019–2024

Only data OFF medication and OFF DBS was assessed.

### Data acquisition

Any data included in this analysis was recorded OFF medication while patients were in a resting-state. For cohort 1, data were acquired postoperatively from the ring contacts of externalized DBS leads between 3 and 6 days after surgery, sampled at 2048 Hz via a TMSi Porti (TMS International, Netherlands) or at 4096 Hz via a TMSi Saga32 (TMS International, Netherlands). Recordings from individual ring contacts were considered as measurements at different depths herein. For cohort 2, data were acquired postoperatively from the ring contacts of externalized DBS leads 1 day after surgery, sampled at 2 kHz via a custom connected MEG system (VectorView, MEGIN), and bandpass filtered between 0.1 and 660 Hz. Likewise, recordings from individual ring contacts were considered measurements at different depths herein. For cohort 3, data were acquired intraoperatively (local anesthesia) from the tips of two independently controlled microelectrodes, sampled at ≥12.5 kHz via two Guideline System GS3000 amplifiers (Axon Instruments, USA). Measurements were taken along the implantation trajectory, whenever a neuron was clearly visible. For cohort 4, data were acquired intraoperatively (general anesthesia) at the tips of 3–5 microelectrodes mounted in a Ben-Gun array, sampled at 20 kHz via an Isis IOM system from Inomed, Germany. Measurements were taken every 0.5 mm, starting 5 mm before the STN’s center for at least twelve consecutive steps. Demographic information is provided in Table 2. For cohort 5, data were acquired intraoperatively (local anesthesia) from the tips of 5 microelectrodes mounted in Ben-Gun array, sampled at 24 kHz via a Leadpoint system from Natus, Germany. Measurements were taken every 1 mm starting 5 mm before the STN’s center for ten consecutive steps. Demographic information is provided in Table 2.

**Table 2:** PD subtypes are tremor/equivalence/rigidity

Cohort	Parkinson’s subtype	DoB	H&Y Med ON	Disease onset	Surgery	Disease duration	Age at surgery
4	16/48/36%	1958 ± 8.3	2.18 ± 0.42	2010 ± 4.83	2021 ± 2.25	10.63 ± 4.84	62.5 ± 8.21
5	20/33/47%	1961 ± 7.8	2.19 ± 0.54	2012 ± 5.1	2021 ± 1.78	9.27 ± 4.31	59.5 ± 7.36

Disease duration is in years.

### Data pre-processing

The data were zero-centered and detrended. A lowpass was applied (cohorts 1 and 2: 299 Hz; cohorts 3/4/5: 4999 Hz), and the data were downsampled (cohorts 1 and 2: 600 Hz; cohorts 3/4/5: 10,000 Hz). The data were manually screened for visually identifiable artifacts, (i.e. movement) and continuous, artifact-free recordings longer than 5 s (60–160 cycles of beta activity) were retained. Whenever multiple trajectory sets with different ring/arc angles were explored, only the final set with the DBS lead as reference point to recover anatomical position was evaluated.

Datasets 4 & 5 were recorded with a weak (−3 dB) hardware high-pass filter applied at the time of the recording with a cutoff at 30 (dataset 4) and 500 Hz (dataset 5). While the low frequency component’s amplitude was attenuated, it was not lost as its amplitude remained several orders of magnitude above the threshold required for successful digital representation. The original signal was restored by applying an inverse filter. Proper signal restoration was visually confirmed (see Supplementary Fig. 1).

### Anatomical reconstruction of microelectrode positions

Using computer tomography (CT) and magnetic resonance images (MRI) available, microelectrode trajectories explored in cohorts 4 and 5 were reconstructed, supporting their analysis. A reference trajectory was recovered via Lead-DBS^52^: The software was used to reconstruct the position of the implanted DBS lead in MNI space, localizing measurements along one microelectrode trajectory. The remaining microelectrode trajectories (up to 4; variable) were reconstructed via parallel projection matching the BenGun array’s shift (2 mm), documentation on explored trajectories (i.e. central and lateral), and the intraoperatively employed ring and arc angles. Subsequently, measurement points along all microelectrode trajectories were determined by combining implantation depths, regularly spaced measurement points, and the structure of the implanted DBS leads. The HybraPD atlas^53^ used to project the STN into the reconstruction frame.

Reconstruction accuracy of DBS-lead positions, and microelectrode measurements was manually verified for each patient (see Supplementary Fig. 2). 3D and 2D visualizations of the anatomical reconstructions of patients from cohort 4 and 5 are available in the supplementary material.

### Beta peak detection

To identify beta peaks, the frequency spectra of all recordings obtained in a single hemisphere were concurrently visualized. Welch’s method (0.25–1 Hz frequency bin width) was employed to project signals into the frequency domain. Individual power spectra were visualized for 1–6, 6–14, 5–45, 10–36, and 15–36 Hz. Separate windows, normalized to their relative maxima, allowed for a highly resolved display, particularly at higher frequencies (see Supplementary Fig. 3). A copy of each hemisphere’s LFP peak screening is provided in the supplementary materials.

### Quantifying LFP peak prevalence

Individual LFP peaks were identified and screened for artifacts. Analyses were conducted manually due to automated approaches inferior performance (30–76% accuracy) and major variance across subjects^54^. Peaks were identified when LFP activity was locally elevated above noise levels in the alpha or beta bands. Given the significant variation in noise across subjects, the large of variety of possible artifacts, and the additive nature in which these present, the noise level was visually identified.

In the next step, artifacts were rejected, this included harmonics (spectrally repetitive effects that occur at distinct intervals, i.e. 3 Hz, 6 Hz, 9 Hz etc.) and peaks with the exact same amplitude across several depths (characteristic of electrical artifacts). Peaks whose spectral center varied by more than 3 Hz were considered distinct phenomena (i.e. low and high beta). Leveraging cohorts’ 3–5 increased data density, peaks were also rejected if they were only observed in a single measurement and never before/thereafter, and in case peaks were expressed across all measurement depths (rejected if physiologically impossible; i.e. 9 mm of continuous STN along a singular trajectory). Finally, employing anatomical data for cohorts 4 & 5, peaks were additionally required to be anatomically within the STN. Of note, any trajectory which did not contain a minimum of three sequential recordings acquired within the STN was marked as invalid, removed from evaluation, and not counted against beta presence or absence. To compensate for potential inaccuracies in the reconstruction process, beta peaks within a 1 mm margin around the STN were considered within the STN.

A classification example is discussed in the Supplementary Materials.

### Single-unit processing

The moving average of a recording was subtracted from the raw signal to delineate single unit activity (see Supplementary Fig. 4). Subsequently, spikes were identified by applying a manually adjustable amplitude threshold. Next, the temporal gaps between individual spikes (inter-spike-interval) were measured. When the inter-spike-interval between spikes was below a specific threshold, spikes were considered part of a single burst. Inter-spike-intervals above the threshold separated individual bursts. This threshold was initially set at 20 ms, the average of within burst firing From these, bursts were delineated by identifying those spikes with an inter-spike-interval below a certain threshold, initialized as the average of within burst firing (~10 ms^55^) and tonic spiking activity rate (~45 ms^56^). To address variability in neuronal activity, this threshold was adjusted between individual recordings in case visual inspection suggested another threshold would result in a more clean separation of individual bursts. As spikes within bursts are more closely tied to the LFP^33^, only those were retained. Via Welch’s method, the spectrum of the within-burst spiking activity was computed.

### Single-unit rating & analysis

To identify beta-bursting neurons, microelectrode recordings were screened for bursting neurons that expressed a beta spike in the neuron’s enveloped power spectrum. From these, we delineated recordings wherein bursting and low frequency peak were robust, expressed for an extended period and attributable to a single unit. These were termed *high fidelity* neurons. Any analyses were performed on both, all and high fidelity neurons exclusively.

Phase amplitude coupling (PAC; direct modulation index^57^) and coherence (directional absolute coherence^58^) between the within burst spiking activity and the LFP were calculated, the latter filtered to the bursting peak frequency ±3 Hz. Magnitude and phase were extracted from the PAC estimate. Magnitude and directionality from the coherence estimate. Neurons with a PAC of greater than 0.1 (scale: 0 to 1) were discarded. Neurons with a PAC of greater than 0.4 were classified as high PAC neurons. A binomial test was used to assess whether neurons’ activity was equally likely to precede and succeed LFP activity.

A classification example is discussed in the Supplementary Materials.

## Supplementary information

Supplementary information

## Data Availability

Data from cohorts 1 to 3 are publicly available^33,46,51^. Data from cohorts 4 & 5 may be shared upon request. Graphical documentations for all LFP/spike evaluations individually, as well as microelectrode reconstructions, are available at zenodo^59^.

## Code availability

Python code written to support this evaluation has been made available at: https://codeberg.org/MaximilianScherer/2026_MS_beta_prevalence.git^60^.
