# Supplementary material for: Inconsistent subthalamic local field potential beta activity amid in- and antiphasic neuronal bursts
Source: NPJ Parkinsons Dis. 2026 Sep 16;12:222. doi: 10.1038/s41531-026-01531-4 (PMC13582961; doi:10.1038/s41531-026-01531-4)
Supplement: Supplementary file 1 [file 41531_2026_1531_MOESM1_ESM.pdf]

Supplementary Materials

Contents

Supplementary Figures and Examples.. 2
Individual LFP and spike assessments, and microelectrode reconstructions...................................... 5
Alpha power prevalence.. 6

Supplementary Figures and Examples

Restoration of low frequency activity in cohorts 4 (panels A1 and A2) and 5 (panels B1 and B2).

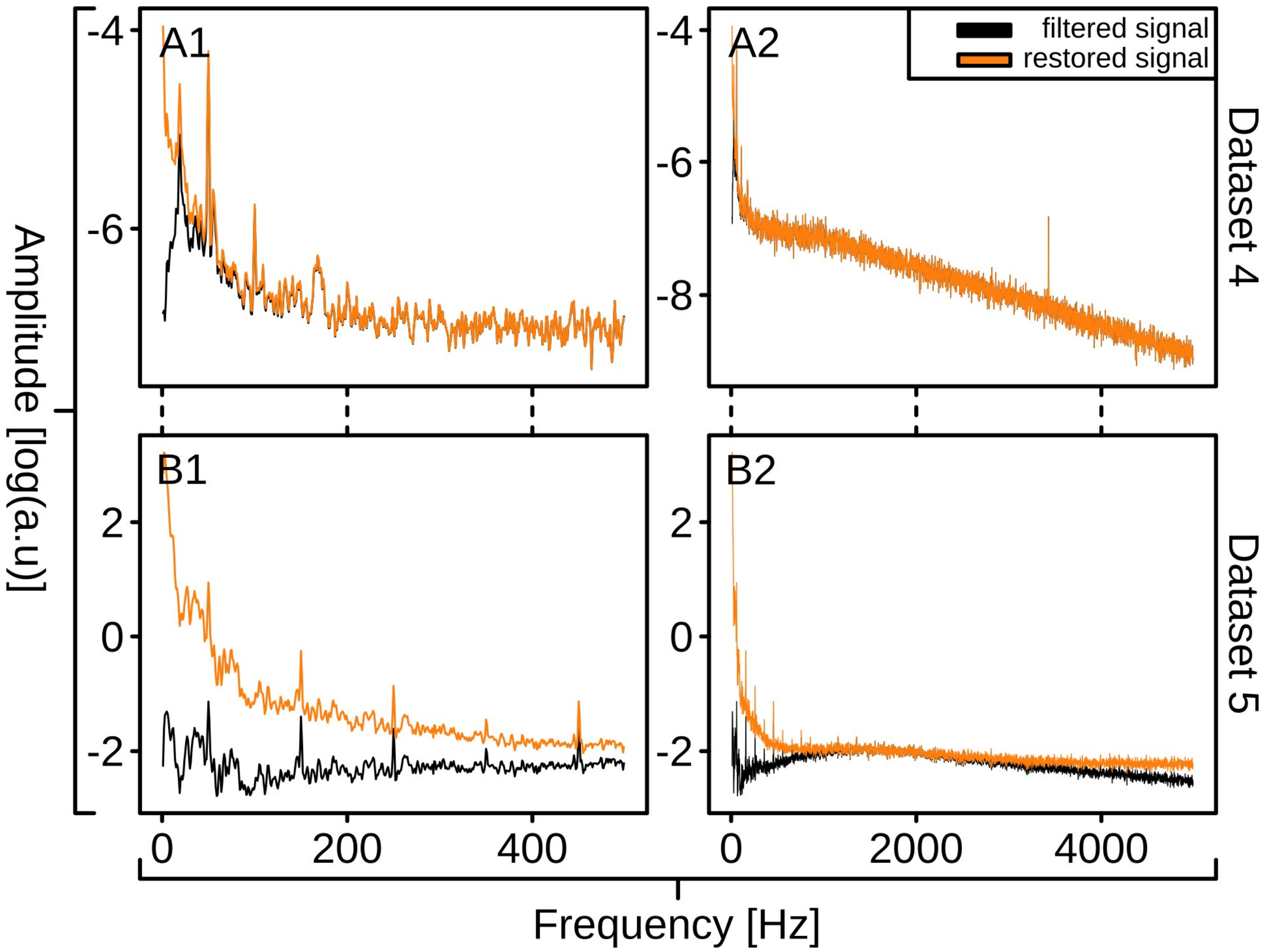

Supplementary Figure 1: Frequency domain signals prior (black) and after (orange) filter reversal. Pink noise (1/f) is restored, as is information in the corresponding frequencies. A1 and A2 show a sample for database 4 and B1 and B2 for database 5.

Microelectrode trajectory localization

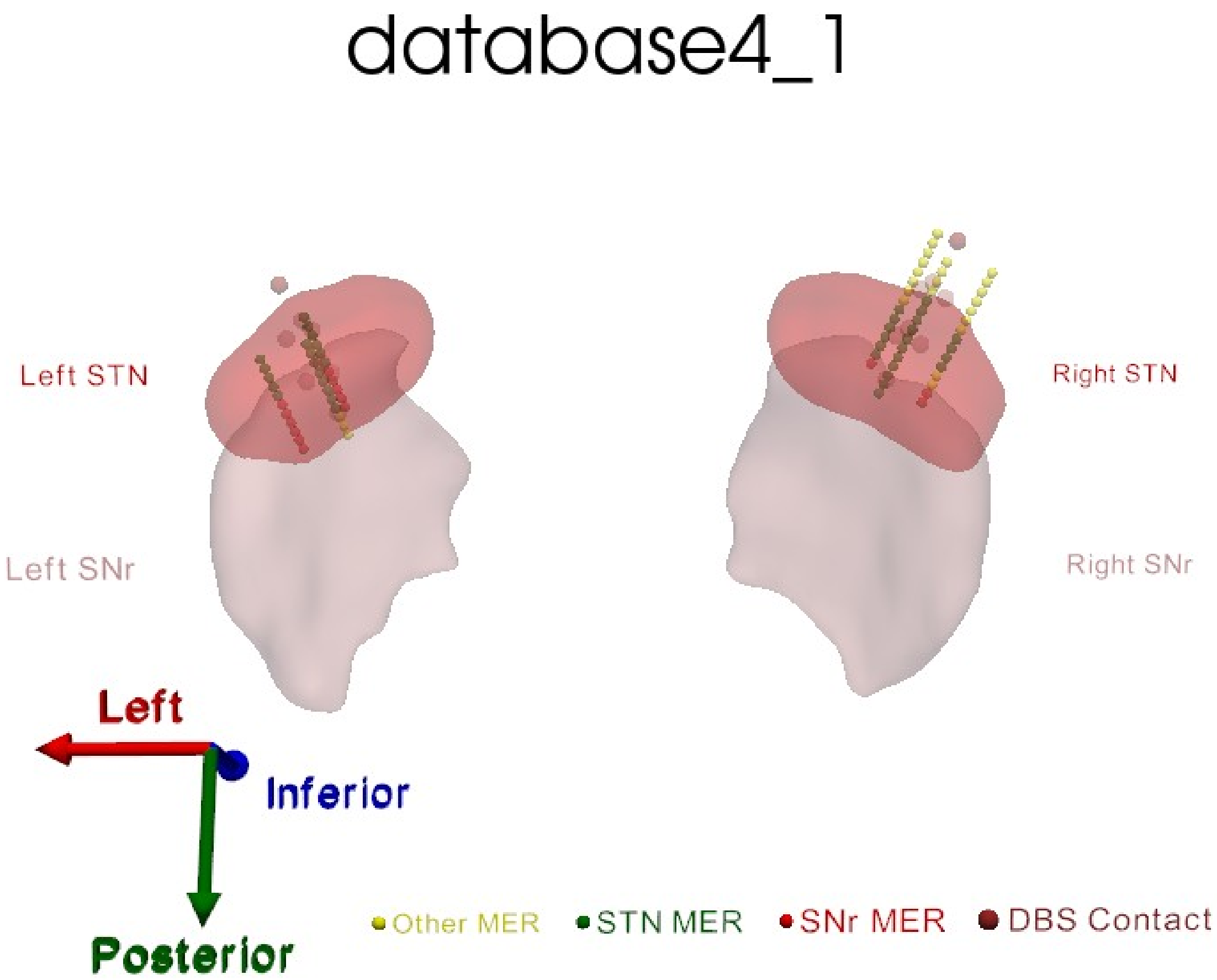

Supplementary Figure 2: Sample 3D visualization of explored microelectrode trajectories from the 1st patient in dataset/cohort 4. STN is red, SNr pink, MER measurement points are indicated as yellow (other region), green (STN), and red (SNr) dots. DBS contacts, which acted as the basis for the reconstruction, are indicated by brown, bigger, dots.

LFP detection tool
Sample of the custom written LFP peak detection tool employed in this investigation.

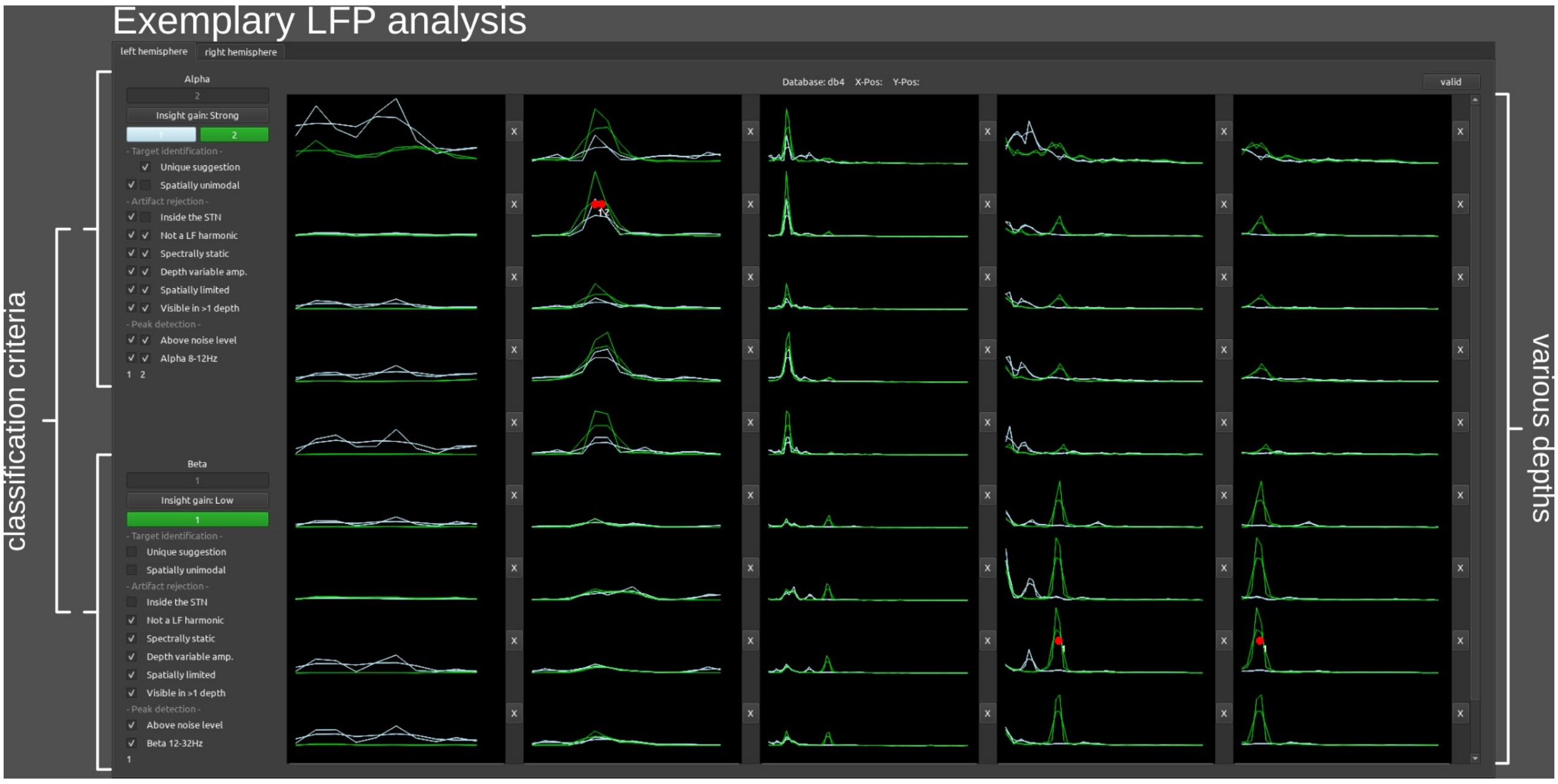

Supplementary Figure 3: Custom UI developed to identify beta LFP peaks.

Supplementary Figure 3 is a screenshot of the LFP analysis tool used to identify peaks in the alpha and beta frequency band. Spectral peaks were identified cross several frequency bands. To be recognized as a valid spectral peak, several criteria had to be me. While the figure above presents a summary, an extensive description of these criteria is provided in the following section, *LFP detection criteria*. Results for all investigated patients are available in the provided ZIP file in the following directories:

- "*lfp_peak_detection/database1/*"
- "*lfp_peak_detection/database2/*"
- "*lfp_peak_detection/database3/*"
- "*lfp_peak_detection/database4/*"
- "*lfp_peak_detection/database5/*"

Criteria for LFP peak selection are described in the following paragraph.

Examples for peak detection
A peak at 15 or 20 Hz would meet this requirement, if its elevation was beyond the noise level in the investigated signal. Given the significant variation in noise across subjects, the large of variety of possible artifacts, and the additive nature in which these present, the noise level was visually identified as automation considering all these characteristics would be highly complex and at the same time very unlikely to be able to correctly deal with unexpected artifacts.

Examples for Artifact rejection
In case a peak's 15 Hz activity was mirrored by activity at 10 Hz and 5 Hz, it was classified as a harmonic distortion. In case it would have been expressed across too many investigated depths (> 8 mm), it would fail to meet the spatially limited requirement. Finally, in case the peak was only observed in a single measurement and not within 2 mm thereafter, it was considered non-reproducible and hence not disease-specific. This criterion was only applied to datasets 3-5 as measurement density in datasets 1 & 2 was too low to discern non-reproducible beta peaks from non-optimally placed DBS leads. As spectral artifacts are quite common and the beta-band LFP commonly extends significantly

Exemplary spike analysis

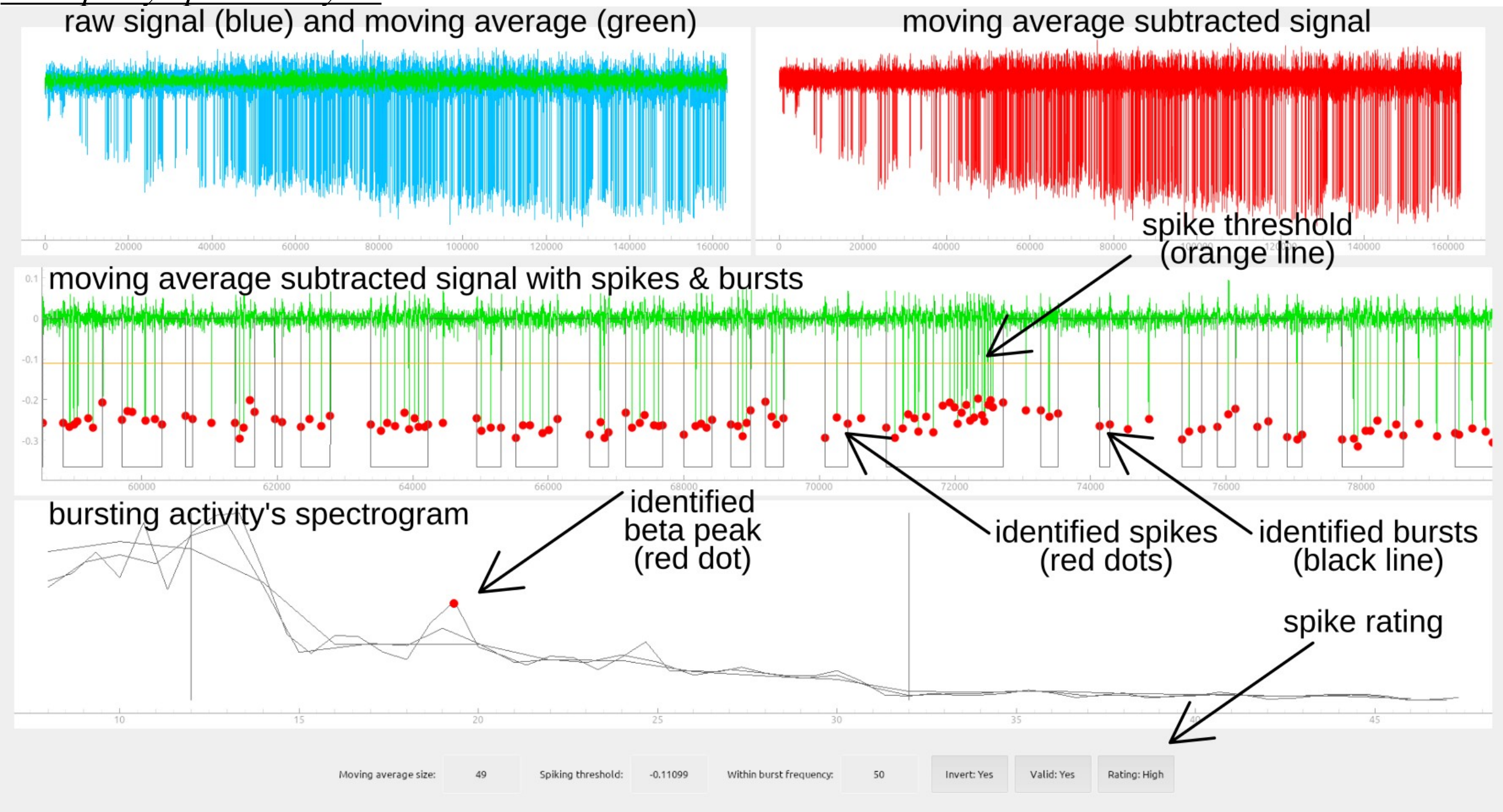

Supplementary Figure 4: Custom UI developed to identify beta bursting neurons.

Results for all positively screened recordings are available in the provided ZIP file available on zenodo (doi.org/10.5281/zenodo.17951550) in the following directories:

- "*spike_burst_detection/database1/*"
- "*spike_burst_detection/database2/*"
- "*spike_burst_detection/database3/*"
- "*spike_burst_detection/database4/*"
- "*spike_burst_detection/database5/*"

Criteria for spiking and burst detection are described in the following paragraph.

Individual LFP and spike assessments, and microelectrode reconstructions

These are available on zenodo (doi.org/10.5281/zenodo.17951550).

Alpha power prevalence
Peaks in alpha power (Figure 5), the control condition, were far less prevalent, as anticipated. Furthermore, variability and artifact presence were higher as well.

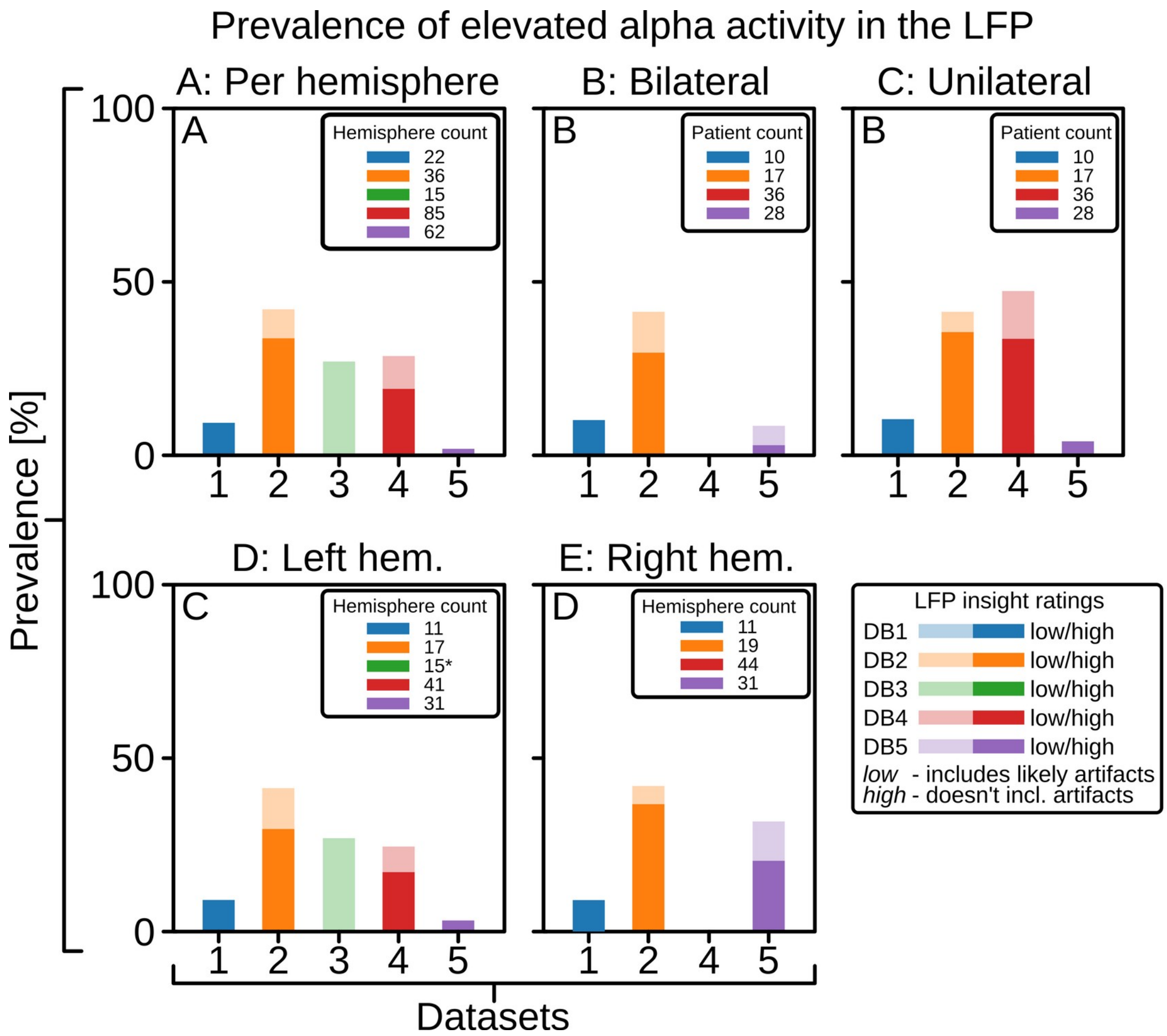

Supplementary Figure 5: Alpha power peak prevalence.
